# INTERSECTIONAL AND BIOSOCIAL PATHWAYS TO COGNITIVE FUNCTION AMONG OLDER HISPANICS AND LATINOS

**DOI:** 10.1093/geroni/igad104.0004

**Published:** 2023-12-21

**Authors:** Catherine Garcia, Lauren Brown

**Affiliations:** Syracuse University, Syracuse, New York, United States; University of Southern California, Los Angeles, California, United States

## Abstract

Hispanics/Latinos are the fastest-growing segment of older adults in the United States (U.S.). They are at high risk for cognitive impairment (CI) and Alzheimer’s disease and related dementias (ADRD). While increased research attention has been given to the socioeconomic and cultural factors that influence the prevention, diagnosis, and care provided to Hispanics/Latinos with CI and ADRD, there are still theoretical and methodological gaps in clarifying the mechanisms and pathways that produce ADRD inequities, including life course and multilevel mechanisms. Given the diverse origins of Hispanics/Latinos in the U.S. – in ethnic origin, birthplace, and current residence – it is crucial to understand how these social conditions get “under the skin” that influence cognitive function in older adulthood. Namely, it is important to unpack how different intersectional positions show different relationships with deprivation that influence cognitive and healthy aging. Thus, this study had two main objectives: (1) to examine the mediating role of physiological (dys)regulation between education and cognition, and (2) to understand for which Hispanic/Latino subgroups the interrelated relationships are stronger/weaker for. Correspondingly, we employed multigroup structural equation modeling to address our study objectives. We used data from the Health and Retirement Study (HRS) to discern and assess the effects between education, physiological factors, and cognitive function at baseline and follow-up by Hispanic/Latino subgroups (e.g., foreign-born Mexicans, U.S.-born Mexicans, island-born Puerto Ricans, foreign-born Cubans, etc.). The results from this study underscore the role of structural interventions and community engagement in reducing and eliminating cognitive health disparities among Hispanics/Latinos.

